# Age Estimation of Faces in Videos Using Head Pose Estimation and Convolutional Neural Networks

**DOI:** 10.3390/s22114171

**Published:** 2022-05-31

**Authors:** Beichen Zhang, Yue Bao

**Affiliations:** Visual Media Laboratory, Department of Information Science, Tokyo City University, Tokyo 1588557, Japan; bao@g.tcu.ac.jp

**Keywords:** age estimation, deep learning, CNN, head pose estimation

## Abstract

Age estimation from human faces is an important yet challenging task in computer vision because of the large differences between physical age and apparent age. Due to the differences including races, genders, and other factors, the performance of a learning method for this task strongly depends on the training data. Although many inspiring works have focused on the age estimation of a single human face through deep learning, the existing methods still have lower performance when dealing with faces in videos because of the differences in head pose between frames, which can lead to greatly different results. In this paper, a combined system of age estimation and head pose estimation is proposed to improve the performance of age estimation from faces in videos. We use deep regression forests (DRFs) to estimate the age of facial images, while a multiloss convolutional neural network is also utilized to estimate the head pose. Accordingly, we estimate the age of faces only for head poses within a set degree threshold to enable value refinement. First, we divided the images in the Cross-Age Celebrity Dataset (CACD) and the Asian Face Age Dataset (AFAD) according to the estimated head pose degrees and generated separate age estimates for images with different poses. The experimental results showed that the accuracy of age estimation from frontal facial images was better than that for faces at different angles, thus demonstrating the effect of head pose on age estimation. Further experiments were conducted on several videos to estimate the age of the same person with his or her face at different angles, and the results show that our proposed combined system can provide more precise and reliable age estimates than a system without head pose estimation.

## 1. Introduction

Age estimation from a facial image has become an important yet challenging problem in many applications, such as human–computer interaction [1,2,3], identification [4], security [5], and precision advertising [6].

**Figure 1:**
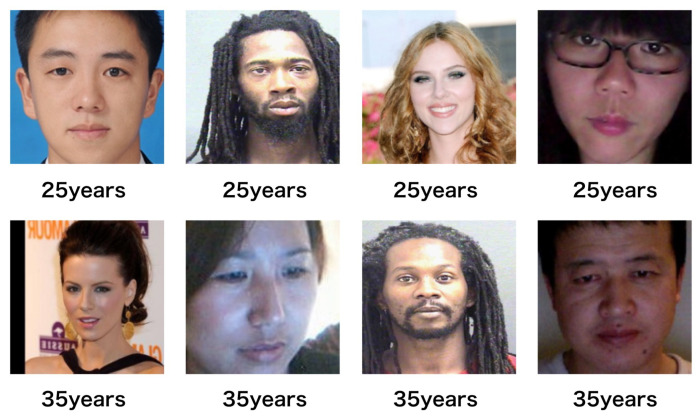
Differences between different people of the same age.

**Figure 2:**
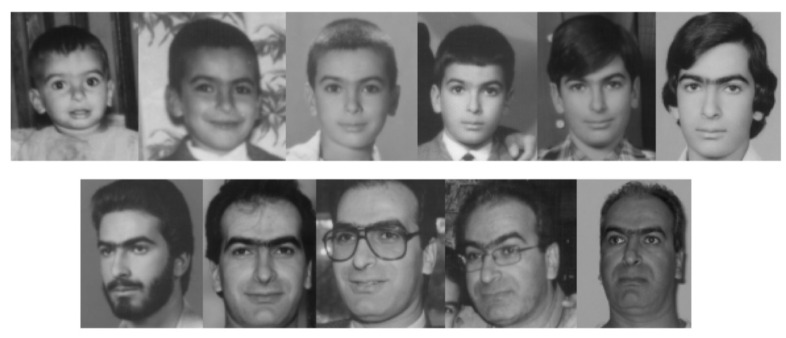
Changes in facial appearance from childhood to adulthood.

Although there have been a great deal of studies on the age estimation issue, the performance of age estimation from facial images is still a huge gap with real-life application demands in terms of both accuracy and stability. The reasons that make age estimation such a challenging problem come from two groups: Objective conditions of the external environment that include illumination, distance, pose, perspective, and expression [7]; physiological conditions of intrinsic features that include ethnicity, gender, and health status [8]. Previous studies carried out a lot of work on the external conditions since the intrinsic features can not be normalized and are therefore difficult to solve. The intrinsic facial features are always inhomogeneous for several reasons: (1) even people of the same age can exhibit enormous variation in facial appearance (Figure 1); and (2) in different periods of age, human faces change differently. For example, children usually have a fast speed of bone growth; on the other hand, adults’ faces change very slowly [9] (Figure 2). Consequently, it is a difficult problem to make an estimator which could predict real age of human face images accurately from such widely diverse appearance factors.

In recent years, deep learning has led to impressive works on various computer vision tasks, including age estimation [10,11,12]. However, all these works have used datasets including only frontal facial images, which cannot adequately reflect the conditions of real-life applications. Different from most facial images in datasets, the head pose may vary greatly in videos or webcam streams, leading to intolerable errors in the estimated age.

In this work, a combined system of age estimation and head pose estimation is proposed to solve the problem of age estimation from faces in videos or webcam streams. First, we use deep regression forests (DRFs) [11] to estimate the age of facial images, which can achieve high precision for frontal facial images. Meanwhile, a multiloss convolutional neural network (CNN) is also utilized to estimate the head pose [13]. Then, we can use the trained system to estimate age and head pose from several videos frame by frame. When using the trained mapping between age and head pose, we set a degree threshold for the head pose and perform age estimation only for frames where the head pose is within this threshold to enable value refinement of the age estimated from the video.

Experiments were conducted in two phases. First, a multiloss CNN was trained on the 300W-LP dataset [14] for head pose estimation. We also divided the Cross-Age Celebrity Dataset (CACD) [15] and the Asian Face Age Dataset (AFAD) [12] based on the estimated head pose angles and trained DRFs separately on the subsets of frontal and nonfrontal images. The results showed that the accuracy of age estimation from frontal facial images was better than that for faces at different angles. Then, we tested the trained models on several videos to estimate the age of the same person with his or her face at different angles. The experimental results demonstrate that our proposed system with a head pose angle constraint achieves a standard deviation of the estimation errors for videos that is smaller than what can be achieved when performing age estimation alone. The results show that our approach improves the precision and reliability of age estimation for faces in videos compared to traditional methods.

This paper consists of five sections. Section 2 introduces some related works of age estimation, Section 3 presents the proposed method with the whole architecture and several details, the experimental results for the proposed method are discussed in Section 4, and a conclusion and a discussion of future work can be found in Section 5.

## 2. Related Work

Deep learning methods are used in age estimation because of their great success in many computer vision tasks. Similar to [16], Yi et al. [17] used CNN for age estimation with the features extracted from different regions of the face, and introduced the mean squared loss as the measurement criterion. Niu. Z et al. [12] noticed the continuous feature of age and trained an ordinal CNN; multiple binary outputs were also used for better performance. Another use of continuous information comes from [10] with multiple binary neural networks; the multiple outputs were aggregated as final result. Ref. [18] used softmax fuction in another way, in which softmax outputs of each neuron were used as weights of age, and a weighted average value was calculated instead of using the softmax classification result directly; the experiment results showed better performance. Multi-task learning methods were used for age estimation in [19,20]; several other facial features were jointly learned and enhanced the performance of each task. Deep regression forests (DRFs) [11] used random regression forests coupled with CNN and obtained better performance.

For age estimation from faces in videos, the most closely related work is the deep age estimation model [21], in which Ji et al. used a CNN with an attention mechanism. Facial features were extracted by CNN then aggregated from features vectors to a single feature by an attention block. They trained the model using a new loss function, leading to better precision and stability across every frame for age estimation.

Another work for age estimation where static and dynamic features can be learned from expressions of face simultaneously in videos is called the spatially-indexed attention model (SIAM) [22]. In this model, Ji Pei et al. employed CNNs to extract the latent appearance features from each frame and then used recurrent networks to process all the features to simulate time dynamics. Furthermore, they used a specifically designed spatially indexed attention mechanism, and all the accentuated facial areas in each frame could be extracted by convolutional layers. A time attention layer was also used to allocate attention weights at each frame. This method focuses on both frames and face areas with important information, resulting in better performance. The relevance between spatial facial areas and time frames, as well as age estimation, can also be revealed.

However, Ji et al. used continuous frames as input data, rather than using a single image, in order to guarantee stability, which increased the computation and made the network more complicated. In addition, to train this model, a new dataset must be collected with labels’ annotation, causing more time consumption. The SIAM method has limitations in terms of which types of facial expression images it can consider; specifically, only smile and disgust databases were used in experiments. Therefore, we want to propose a new approach that can be trained with all types of facial images from existing databases using a single image as input to make the age estimation easy and feasible.

## 3. Proposed Method

In this section, each step of the system flow will be explained in detail.

### 3.1. Datasets

**Figure 3:**
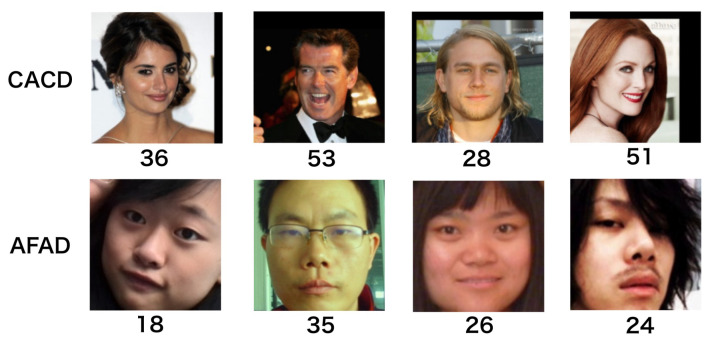
Examples from Cross-Age Celebrity Dataset [15] and Asian Face Age Dataset [12]. The number below each image is the ground truth age of the subject.

In these experiments, we used two datasets containing different racial groups for age estimation training and one dataset for head pose estimation. Figure 3 depicts exemplar images from each dataset for age estimation.

**Cross-Age Celebrity Dataset:** The CACD dataset, released in 2014 by the University of Maryland Computer Science Department [15], is a large-scale dataset for face recognition and retrieval across ages. It contains 163,446 images of 2000 celebrities. Images were collected by search engines using keywords of the celebrity’s name and a year (2004–2013). The age of the celebrity on the image can be estimated by simply subtracting the year of birth from the year the photo was taken. There are training, validation, and testing parts of the dataset and the training part is very noisy. Therefore, in our experiments, we only used a cleaned subset which was hand-selected to 18,171 photographs. For evaluation, the dataset was randomly divided into 85% for training and 15% for testing.

**Asian Face Age Dataset:** AFAD [12] released this in 2016 for age recognition, containing 164,432 images of faces with accurate age and gender labels. As the Asian Face Age Dataset (AFAD), all of the images from the dataset are Asian faces. AFAD was built by collecting selfie photos from the Renren social network (RSN). Not only do a large number of Asian students from middle school to graduate school use RSN frequently, but plenty of graduated students also use RSN to contact their classmates. Therefore, the ages of RSN users span a wide range from 15 to more than 40 years old. We used a subset of AFAD with about 60k images of people from 18 to 39, and the subset was balanced for training. For evaluation, the dataset was randomly divided into 85% for training and 15% for testing.

**300W across Large Poses:** The 300W dataset [14] uses 68 landmarks to standardize multiple alignment databases, including AFW [23], LFPW [24], HELEN [25], IBUG [26], and XM2VTS [27]. A face profiling method was applied to 300W, and about 60 thousand pose data were extracted (about 1800 in IBUG, 5200 in AFW, 16,000 in LFPW, and 37,000 in HELEN; the data from XM2VTS were not in use). All the data were flipped, and therefore multiplied to 120 thousand. The resulting dataset is called 300W-LP (300W across Large Poses).

### 3.2. Face Alignment

Detection performance will be changed as well as the surroundings of the face change. The different types of face alignments could result in additional performance changes. An ideal facial image should have similar size, with front view, face centering, and the face alignment normalized with fixed location and cleaned background. Therefore, we chose the multi-task cascaded convolutional network (MTCNN) [28] face detector to obtain the face from an image. In order to minimize the impact of surrounding pixels, we resized all the images to 256×256 and made a random crop to 224×224. The crop process makes the face randomly located at a different position in the image, regardless of the originating data. This approach can improve the robustness of our model to figure out the problem of various scenes with different face alignments. The 224×224 pixel image also fits the input size of VGG-16 network.

### 3.3. Age Estimation

**Figure 4:**
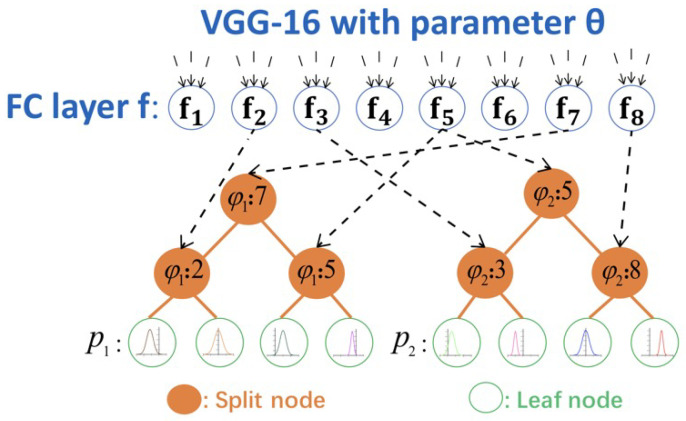
Illustration of deep regression forests.

Figure 4 shows a diagram of a DRF [11]. A CNN combined with deep regression forests is introduced in this work and estimates the real age from facial image. The model is trained on facial image datasets with known ages and face landmarks as labels. The training process in this paper begins with the pretrained weights from the ImageNet dataset, as with the same model used in [29]. Then, the CNN is fine-tuned on the two target datasets used for age estimation. The fine-tuning process makes the CNN obtain the features, distribution, and bias of each dataset and optimizes the performance.

The upper blue circles represent the output neurons from the CNN defined by the function **f** with parameter θ. All these neurons come from the last fully-connected (FC) layer of VGG-16. The middle orange circles represent the split nodes and the bottom green circles represent the leaf nodes of deep regression forests. $\varphi_{1}$ and $\varphi_{2}$ represent the index functions of each tree. The black dashed arrows point out the correspondence from the split nodes of each tree to the neurons of VGG-16 FC layer. Each neuron may correspond to the split nodes of different trees. Each tree has its own distribution π for its leaf node (represented by the distribution curves on the leaf nodes). The final output for the whole forest can be calculated as the mix of the predictions of the individual trees. The parameters f(·;Θ) and π will be trained simultaneously end-to-end.

**VGG-16:** The VGG-16 CNN architecture was selected since, first, the architecture of VGG-16 is deep, representing high performance, but also manageable, representing expandability; secondly, Russakovsky et al. have achieved impressive work [30] with the VGG-16 model on the ImageNet challenge; and thirdly, pretrained models for classification of VGG-16 are publicly available, which can accelerate the process of training. VGG-16 network is much deeper than previous architectures, for example, AlexNet [31], specifically, consisting of 13 convolutional layers and 3 FC layers. It is characterized by a number of 3×3 filters with convolution kernel and the stride of filters are set to 1; within comparison, AlexNet has much larger filters (up to 11×11), and the stride of filters are set to 4. Therefore, each convolution filter from VGG-16 has simpler geometry, but the increased depth also allows much more complexity.

**Deep Regression Tree:** DRFs are combination of several deep regression trees. For each tree, three are input–output pairs ${\left\{x_{i},y_{i}\right\}}_{n=1}^{N}$, in which $x_{i}\in R^{D_{x}}$ and $y_{i}\in R$. A deep regression tree model describes the mapping relationships from input to output over CNNs connect with a regression tree. A deep regression tree T has a number of split nodes N and leaf nodes L. To be specific, an input $x_{i}$ will be passed to the left or right node relative to one node, which will be decided by each split node n∈N; while for a leaf node ℓ∈L, it can be described by a Gaussian distribution, where $p_{\ell }\left(y_{i}\right)$ represents the mean and $\mu_{l}$ represents the variance $\sigma_{l}^{2}$ of Gaussian distribution.

**Split Node:** A split node is associated with a splitting function $S_{n}\left(x_{i};\Theta \right):x_{i}\to \left[0,1\right]$, which is parameterized by Θ—the parameters of CNNs. Normally, this splitting function is defined as $s_{n}\left(x_{i};\Theta \right)=\sigma \left(f_{\varphi (n)}\left(x_{i};\Theta \right)\right)$, where σ(·) represents the sigmoid function, φ(·) represents an index function to point out the φ(n) element of $f(x_{i};\Theta )$ consistent with the split node *n*, and $f(x_{i};\Theta )$ are the learned deep features. Figure 4 illustrates the simple diagram of the DRFs, where $\varphi_{1}$ and $\varphi_{2}$ represent the index function of each tree. For a given $x_{i}$, the probability of reaching the leaf node l can be calculated as

(1) $$\omega_{\ell }\left(x_{i}|\Theta \right)=\prod_{n\in N}^{}s_{n}{\left(x_{i};\Theta \right)}^{\left[\ell \in L_{n_{l}}\right]}{\left(1-s_{n}\left(x_{i};\Theta \right)\right)}^{\left[\ell \in L_{n_{r}}\right]}$$

Here, $L_{n_{l}}$ and $L_{n_{r}}$ are the sets of leaf nodes belonging to the subtrees $T_{n_{l}}$ and $T_{n_{r}}$. Subtree $T_{n_{l}}$ means that the root of the tree is the left children $n_{l}$ of node *n* and $T_{n_{l}}$ means that the root of the tree is the right children $n_{r}$ of node *n*.

**Leaf Node:** Consider a tree T; for each input $x_{i}$, an ℓ∈L leaf node represents a predictive distribution on $y_{i}$, denoted by $p_{\ell }\left(y_{i}\right)$. Specifically, there we assumed that $p_{\ell }\left(y_{i}\right)$ is in obedience to the Gaussian distribution: $N(y_{i}|\mu_{l},\sigma_{l}^{2})$. Therefore, the final distribution with the conditional probability of $y_{i}$ on $x_{i}$ can be calculated by averaging the probability of the route to each leaf node:

(2) $$p_{T}\left(y_{i}|x_{i};\Theta ,\pi \right)=\sum_{\ell \in L}^{}\omega_{\ell }\left(x_{i}|\Theta \right)p_{\ell }\left(y_{i}\right)$$

where Θ are the parameters from CNNs and π are the distribution parameters $\left\{\mu_{l},\sigma_{l^{2}}\right\}$. This distribution can be considered as a mixed distribution, in which $\omega_{\ell }\left(x_{i}|\Theta \right)$ are the mixing coefficients and $p_{\ell }\left(y_{i}\right)$ represents the Gaussian distributions at the $\ell^{t}h$ leaf node. The π has different value for each tree; therefore, $\pi_{k}$ is used with the corresponding index in the subsequent part.

**Deep Regression Forests:** Deep regression forests are combinations of several deep regression trees, $F=\{T_{1},\ldots ,T_{n}\}$; the final output distribution of prediction can be calculated by an input $x_{i}$, as the average of all trees:

(3) $$p_{F}\left(y_{i}|x_{i},\Theta ,\Pi \right)=\frac{1}{N}\sum_{n=1}^{N}p_{T_{n}}\left(y_{i}|x_{i},\Theta ,\pi_{n}\right)$$

where *N* represents the total number of trees and $\Pi =\{\pi_{1},\ldots ,\pi_{N}\}$. $p_{F}\left(y_{i}|x_{i},\Theta ,\Pi \right)$ represents the possibility when the *i*th input yields output of $y_{i}$.

### 3.4. Head Pose Estimation

In most works on predicting head pose using convolutional networks, the easiest way is using a mean squared error loss, and the output angles of head pose have been regressed directly. However, this approach fails to meet adequate performance requirements on the dataset we wish to use for age estimation.

**Figure 5:**
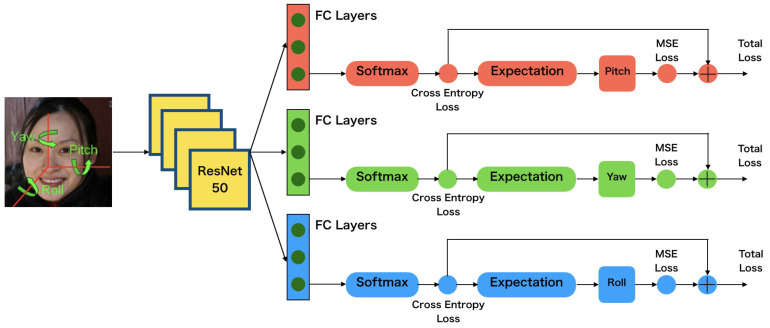
CNN with combined mean squared error and cross-entropy losses.

Therefore, we adopted Ruiz’s method [13], in which deep multiloss CNNs are trained for head pose estimation with satisfactory accuracy. The ResNet50 network [32] was introduced for head pose estimation and three losses are used for three angles separately. There are two parts of each loss: the mean squared error regressed directly and the cross-entropy loss from classification of pose. There are three FC layers being used for three angles and sharing the previous parts of the network. By adopting additional cross-entropy losses from classification, we constructed three signals to be backpropagated to improve the learning process. The predictions of three output angles were computed as the final head pose results. The details of the architecture are shown in Figure 5.

**Mean Absolute Error:** To evaluate the performance of different age estimation algorithms, as a criterion of measurement for age estimation algorithms, mean absolute error (MAE) metric is used for the estimation. By calculating the average absolute error between the precited age and the ground truth age, the defining equation of MAE is

$$MAE=\frac{1}{K}\sum_{i=1}^{K}\left|\tilde{x_{i}}-x_{i}\right|$$

where *K* is the number of samples, $x_{i}$ is the ground truth age of the *i*-th sample, and $\tilde{x_{i}}$ is the predicted age of the *i*-th sample. A small MAE represents great performance of age estimation.

## 4. Experiments

In the following section, the implementation details of experiments are presented along with their quantitative and qualitative results. It concludes with a discussion on the findings.

### 4.1. Implementation Details

For each experiment, we used the existing weights for VGG16 as the initial value from ImageNet. The training parameters of the neural network are listed as follows: the batch size of training data is 64, the ratio of dropout layer is set to 0.5, the stochastic gradient descent (SGD) is used as gradient descent method, the learning rate is set to 0.2 as an initial value, and reduces by half per 5k iterations. The training parameters of the regression forests are listed as follows: the number of trees is set to 4, the depth of each tree is set to 5, the number of output unit is set to 64, the value of leaf node will be updated per 10 iterations, and the prediction result from leaf nodes will be updated per 30 iterations. This model is then fine-tuned with CACD and AFAD for age estimation. ResNet50 was trained on the 300W-LP dataset for head pose estimation, and the training parameters of the ResNet50 are listed as follows: the Adam optimization is used as gradient descent method, and the learning rate is set to $10^{-5}$ with $\beta_{1}=0.9$, $\beta_{2}=0.999$, and $\epsilon =10^{-8}$.

During the training phase, the training data are split as follows: 80% for training and 20% for validation. The training process will be aborted early when the model has been overfit on the validation set. The models were trained on Nvidia GTX 1080 GPUs.

### 4.2. Results and Comparison

First, a multiloss CNN was trained on the 300W-LP dataset for head pose estimation. Subsequently, we divided the images into AFAD and CACD based on the estimated head pose angles and trained DRFs separately on the subsets of frontal and nonfrontal images. Then, we trained DRFs on several subsets of CACD and AFAD with different threshold. Finally, we tested the models on two facial video datasets to estimate the age of the same person with his or her face at different angles and compared the results with those of previous methods. The same network structure and training strategy were used to ensure fair comparisons.

#### 4.2.1. Head Pose Estimation

We trained the adopted multiloss CNN on the 300W-LP dataset in order to make the head pose estimation for age estimation. To verify the performance of the head pose estimation method, we tested the model on a subset of 300W-LP called AFLW2000 [14] which have images cropping around the face area with small size. The AFLW2000 dataset have marked ground truth landmarks; therefore we compared our method with it and other methods, such as commonly used detectors FAN [33] and Dlib [34]. The quantitative results can be seen in Table 1. Although our method is not the best, it is better than traditional detectors and is suitable for our combined system.

#### 4.2.2. Testing on Facial Image Datasets

**Figure 6:**
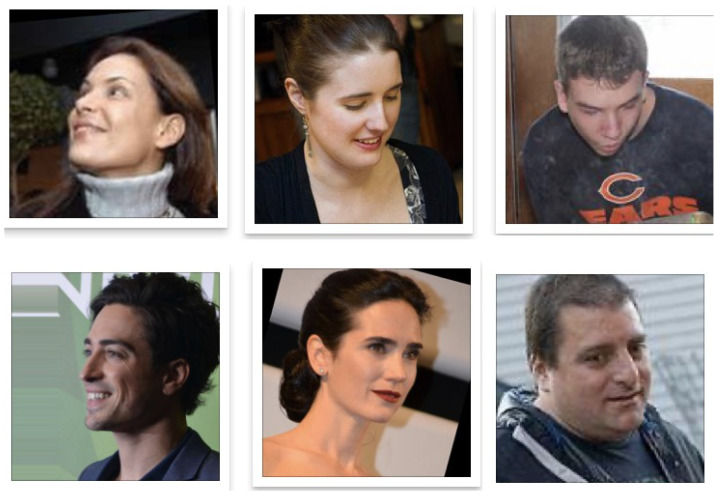
Examples of nonfrontal facial images.

In this section, the performance of DRFs for age estimation based on frontal and nonfrontal facial images is presented. The frequently used AFAD and CACD datasets, representing Asians and Europeans, respectively, were used in this experiment. We used the trained multiloss CNN to estimate the head poses in both datasets. For each facial image, three rotational angles were estimated, one on each axis. We set 30 degrees as the threshold for the sum of the three angles, and images with head pose angle estimates summing to more than 30 degrees were defined as nonfrontal images. Figure 6 depicts exemplar images of nonfrontal facial images from the datasets.

Based on the estimated angles, AFAD was divided into frontal and nonfrontal subsets consisting of 53,983 and 5361 images, and CACD was divided into frontal and nonfrontal subsets consisting of 15,145 and 3026 images, respectively. Both subsets were randomly split into training/test (85%/15%) sets, and the training process was repeated five times with different random separation; the final outcome is the average of five times’ outputs. The quantitative results are summarized in Table 2. The results show that the accuracy of age estimation from frontal facial images is significantly better than that for nonfrontal images.

#### 4.2.3. Testing with Different Threshold

In this section, we separate the CACD and AFAD dataset into several subsets with different thresholds of the head pose degrees. Based on the estimated angles, we set the threshold from 50 degrees to 10 degrees with step of 10 degrees; when the threshold becomes more strict, the number of samples of head pose degree within the threshold become smaller. The correspondence between the threshold and the number of samples, as well as the performance of age estimation, are summarized in Table 3. When the threshold is smaller than 30 degrees, the number of samples reduces rapidly but the performance of age estimation is almost unchanged. Therefore, we chose 30 degrees as the threshold to obtain the best trade-off between performance and number of samples.

#### 4.2.4. Testing on Facial Video Datasets

**Figure 7:**
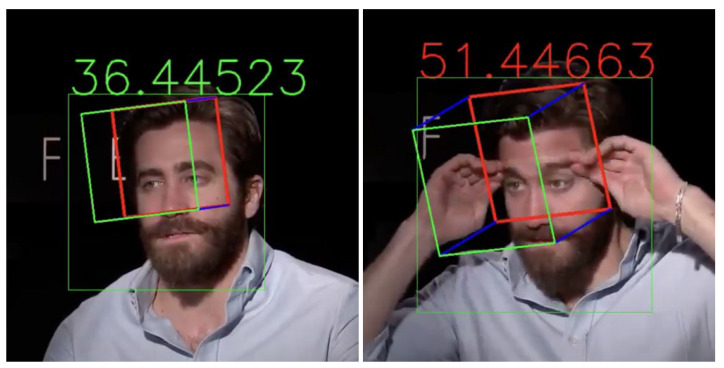
Examples from the facial video datasets with age and head pose estimates. The numbers represent the predicted age. Green and red colors indicate that the sum of the head pose rotational angles is less than and greater than 30 degrees, respectively.

Two new facial video datasets were constructed to evaluate our model in terms of age estimation performance. We collected 18,282 and 18,944 frames from two twelve-minute facial videos of Asian and European subjects, respectively. It should be noted that each facial video dataset was collected from the same person, and these datasets were used only for evaluating the age estimation models; currently, there is no facial video dataset available to be used for training the whole model. We first trained DRFs on AFAD and CACD, representing Asians and Europeans, respectively. Then, we tested the two trained models on the facial video datasets with simultaneous head pose estimation. Examples of the test images are shown in Figure 7. We performed age estimation only for faces with head poses within 30 degrees, and we compared the results with the results for all images without head pose restrictions. Several other models were also trained on AFAD and CACD and then tested on the facial video datasets for more comprehensive comparisons.

We trained a DRF on AFAD and tested the model on the Asian video dataset with head pose restrictions. We also trained a DRF on CACD and tested the model on the European video dataset with head pose restrictions. We compared the results of our method with those of other outstanding age estimation models, and the quantitative results are summarized in Table 4. All models were trained on AFAD and CACD with the same training strategy to ensure fair comparisons. On the task of facial video estimation, our method achieves the best MAE, 5.12, of the Asian facial video dataset and the best MAE, 5.56, of the European facial video dataset. The variance is reduced by 0.62 on the Asian facial video dataset and 1.53 on the European facial video dataset compared to the best existing method. From the results, although the accuracy and performance are different on different datasets, our proposed method can achieve better MAE and variance compared to other methods.

## 5. Conclusions

In this paper, a combined system of age estimation and head pose estimation is proposed to solve the problem of age estimation based on faces in videos or webcam streams, where different head poses may lead to intolerable errors on the estimated ages. Experimental results show that with a head pose restriction such that age estimation is performed only for facial images with head poses within a specified degree threshold to ensure value refinement, our method achieves promising improvements in accuracy and stability for age estimation from video.

The main contributions of this paper are as follows: (1) We are the first to couple age estimation and head pose estimation for age estimation in videos; (2) our method shows significantly improved performance in age estimation on facial video datasets compared to other state-of-the-art methods in terms of both accuracy (MAE) and variance.

However, we only tested our method on two datasets and there might be some video that does not contain any frames that meet our frontal view criteria. In future work, we would collect and annotate more facial images from videos and create a new database including more people. The new database could be trained with our method and obtain more reliable and robust results. We would also attempt to calibrate the nonfrontal samples, instead of just not using them, to make our system widely available. 

## Figures and Tables

**Table 1:** Mean average error of Euler angles across different methods on the AFLW2000 dataset.

Methods	Yaw	Pitch	Roll	Average
Dlib [34]	23.153	13.633	10.545	15.777
Fan [33]	6.358	12.277	8.714	9.116
CPAM [35]	1.479	1.804	1.869	1.697
**Multiloss CNN**	**6.470**	**6.559**	**5.436**	**6.155**
Ground truth landmarks	5.924	11.756	8.271	8.651

**Table 2:** Performance (MAE) comparison on the frontal and nonfrontal subsets of AFAD [12] and CACD.

Subset	AFAD	CACD
Frontal	3.73	4.59
Nonfrontal	4.97	5.65

**Table 3:** Performance (MAE) and image numbers comparison on the AFAD [12] and CACD with different threshold.

Threshold (Degree)	AFAD		CACD	
	MAE	Number	MAE	Number
50	3.97	59,173	4.87	18,023
40	3.84	57,232	4.70	16,842
30	3.73	53,983	4.59	15,145
20	3.72	36,748	4.58	10,398
10	3.71	18,753	4.58	7569

**Table 4:** Accuracy (MAE) and variance results for comparison with state-of-the-art methods on the Asian and European facial video datasets.

Method	Asian		European	
	MAE	Variance	MAE	Variance
AlexNet [31]	6.19	6.92	6.93	7.15
DEX [18]	6.72	8.65	7.17	8.22
DRF [11]	5.96	4.12	6.39	5.84
**Our method**	**5.12**	**3.50**	**5.56**	**4.31**

## Data Availability

Links to datasets used in this paper. CACD: https://bcsiriuschen.github.io/CARC/. AFAD: https://afad-dataset.github.io. 300W-LP: http://www.cbsr.ia.ac.cn/users/xiangyuzhu/projects/3DDFA/main.htm (accessed on 17 April 2021).

## Abbreviations

The following abbreviations are used in this manuscript:

DRFs	Deep regression forests
CACD	Cross-Age Celebrity Dataset
AFAD	Asian Face Age Dataset
CNN	Convolutional neural network
SIAM	Spatially-indexed attention model
FC	Fully-connected
MAE	Mean absolute error
SGD	Stochastic gradient descent
